# A Systematic Review of Economic Evaluations of Health-Promoting Food Retail-Based Interventions

**DOI:** 10.3390/ijerph18031356

**Published:** 2021-02-02

**Authors:** Huong Ngoc Quynh Tran, Emma McMahon, Marj Moodie, Jaithri Ananthapavan

**Affiliations:** 1 Deakin Health Economics, School of Health and Social Development, Institute for Health Transformation, Deakin University, Geelong, VIC 3217, Australia; marj.moodie@deakin.edu.au (M.M.); jaithri.ananthapavan@deakin.edu.au (J.A.); 2 Global Obesity Centre, Institute for Health Transformation, Deakin University, Geelong, VIC 3217, Australia; 3 Wellbeing and Preventable Chronic Disease Division, Menzies School of Health Research, Charles Darwin University, Darwin, NT 0811, Australia; Emma.McMahon@menzies.edu.au

**Keywords:** food retail intervention, economic evaluation, healthy diet, obesity prevention

## Abstract

Background: While the number of retail interventions with impacts on diet- and/or health-related outcomes is increasing, the economic evaluation literature is limited. This review investigated (i) the cost-effectiveness of health-promoting food retail interventions and (ii) key assumptions adopted in these evaluations. Methods: A systematic review of published academic studies was undertaken (CRD42020153763). Fourteen databases were searched. Eligible studies were identified, analysed, and reported following the Preferred Reporting Items for Systematic Reviews and Meta-Analysis (PRISMA) guidelines. Results: Eight studies that evaluated 30 retail interventions were included in the review. Common outcomes reported were cost per healthy food item purchased/served or cost per disability-adjusted life year (DALY) averted. Four studies undertook cost-utility analyses and half of these studies concluded that retail interventions were cost-effective in improving health outcomes. Most studies did not state any assumptions regarding compensatory behaviour (i.e., purchases/consumption of non-intervention foods or food purchases/consumption from non-intervention settings) and presumed that sales data were indicative of consumption. Conclusion: The cost-effectiveness of retail-based health-promoting interventions is inconclusive. Future health-promoting retail interventions should regularly include an economic evaluation which addresses key assumptions related to compensatory behaviour and the use of sales data as a proxy for consumption.

## 1. Introduction

There are well-established relationships between unhealthy diets and chronic non-communicable diseases (NCDs) (i.e., cardiovascular disease, type 2 diabetes, and various types of cancer) [1,2,3,4,5]. Globally, in 2017, approximately 11 million deaths and the loss of 225 million disability-adjusted life years (DALYs) were attributable to dietary risks [1]. Population food consumption is heavily influenced by the food environment, including the food retail environment [6,7]. Living in areas with a high density of unhealthy food retail outlets has been associated with populations with less healthy dietary behaviours and higher body mass index (BMI) [7,8,9,10]. Food retailers are the main source of the foods consumed by households in high-income countries [11,12]. Given the unique role of the food retail system in shaping population diet, interventions to encourage healthier food purchases in food retail settings have great potential to influence current consumption patterns [11].

Over the last decade, research targeted at improving the healthiness of the food retail environment has increased in countries such as the USA, The Netherlands, and Australia [11,13]. Retail interventions are diverse in terms of the type of retail setting, target population, intervention design, and outcome of interest [11,13,14,15]. These interventions include single or multiple strategies to increase the accessibility, availability, and affordability of healthy food options and in-store nutrition information [11,13,15]. The synthesis of the evidence of effectiveness of food retail interventions has found that these interventions have positive impacts on diet-related outcomes, including food purchases, dietary intake, and overall health [13,14,15]. Whilst the published literature related to the effectiveness of healthy food retail interventions is growing [11,13,16], the decision to invest in these interventions requires evidence related to the their economic credentials [17]. Results from economic evaluations can provide an indication of whether an intervention provides good “value for money”. Currently there are no studies that have synthesised the evidence of cost-effectiveness of food retail interventions.

Recent systematic reviews of food retail interventions have found that despite the increasing number of studies, there are limitations in the evidence base [11,16,17]. The diversity of study designs and measurement tools utilised limits the synthesis and generalisability of findings [15]. The primary outcome measure is generally the purchase of the targeted foods [13,15]. However, to have an impact on health, overall consumption needs to change [6]. In addition, there is little evidence on how the increased purchase of targeted healthy foods impacts the purchase of less healthy foods, or foods outside specific product categories [16]. These limitations of the evidence base mean that assumptions are required when modelling the cost-effectiveness of retail interventions in improving overall health outcomes.

The aim of this systematic review is to: (i) assess the evidence of cost-effectiveness of food retail interventions to improve diet-related health outcomes; and (ii) identify the key assumptions used to conduct economic evaluations of food retail-based interventions.

## 2. Methods

### 2.1. Search Strategy

This review was conducted and reported following the Preferred Reporting Items for Systematic Reviews and Meta-Analysis (PRISMA) guidelines [18]. A completed PRISMA checklist is included in Table S1. The protocol was registered on PROSPERO (CRD42020153763). The search strategy, which included Medical Subject Headings (MeSH) terms, was developed by HT and JA and revised with a subject-specific librarian (Tables S2 and S3). The search strategy and the inclusion criteria incorporated three concepts: (1) study design—(i) full economic evaluations, which reported both intervention costs and outcomes (i.e., cost-effectiveness analysis (CEA), cost-utility analysis (CUA), cost-benefit analysis, and cost minimisation analysis) and (ii) reviews of economic evaluations; (2) intervention setting—food retail; and (3) outcomes—diet or health. The search strategy was modified and conducted across 14 electronic databases (EMBASE^®^, Scopus, Web of science, Academic Search Complete; Business Source Complete; CINAHL Complete; EconLit; Global Health; Health Business Elite; Health Policy Reference Center; Health Source: Nursing/Academic Edition; MEDLINE Complete; PsycINFO; and SocINDEX with Full Text). The search was undertaken by HT in August 2019. Reference lists of included studies were screened to identify other potentially relevant articles. There was no restriction on publication year; however, the search was limited to publications in English.

### 2.2. Article Selection

After duplicates were excluded in Endnote [19], the remaining articles were uploaded to Covidence [20] for initial title and abstract screening followed by full text screening. Screening was conducted independently by two reviewers (HT and EM), with conflicts resolved by a third reviewer (JA).

### 2.3. Data Extraction and Synthesis

A standardised data extraction template was developed based on the Consolidated Health Economic Evaluation Reporting Standards (CHEERS) checklist [21]. Key study characteristics by retail setting (i.e., supermarkets, remote community stores, restaurants and fast food stores, school cafeterias, worksite cafeterias and vending machines) that were most relevant to the review objectives, (country, target population, evaluation type, study design, perspective, time horizon, reference year, discount rate, currency, intervention and comparator, intervention cost(s), intermediate intervention effect(s), outcome(s) of interest, and incremental cost-effectiveness ratio (ICER)) were extracted. All costs reported in foreign currency were converted to Australian dollars using Purchasing Power Parity for the reference year reported in the study and then inflated to 2020 values using the Gross Domestic Product Implicit Price Deflator [22,23,24]. Data extraction was completed independently by two reviewers (HT and EM) and verified by a third reviewer (JA).

The logic pathway and the key assumptions reported or required to translate outcomes in retail settings to intermediate intervention effects and long term health outcomes were analysed for each study and synthesised based on the study design (trial-based analyses or modelled evaluations).

### 2.4. Quality Assessments

The quality of each included economic evaluation was assessed against the 24 items of the CHEERS checklist [21], and the compliance of each study was recorded as a percentage. If the included study fully satisfied an item on the CHEERS checklist, one full point was awarded for that item. There were no partial points awarded. Where the item was not applicable to the study, that item was excluded when the compliance percentage was calculated. Although the CHEERS checklist only assesses the reporting quality of economic evaluations, it was used as a proxy to assess the methodological quality of the included studies [21]. Studies were not excluded on the basis of the quality assessment. Two independent reviewers (HT and EM) undertook the assessment, with disagreements resolved by a third reviewer (JA).

## 3. Results

### 3.1. Search Results

**Figure 1:**
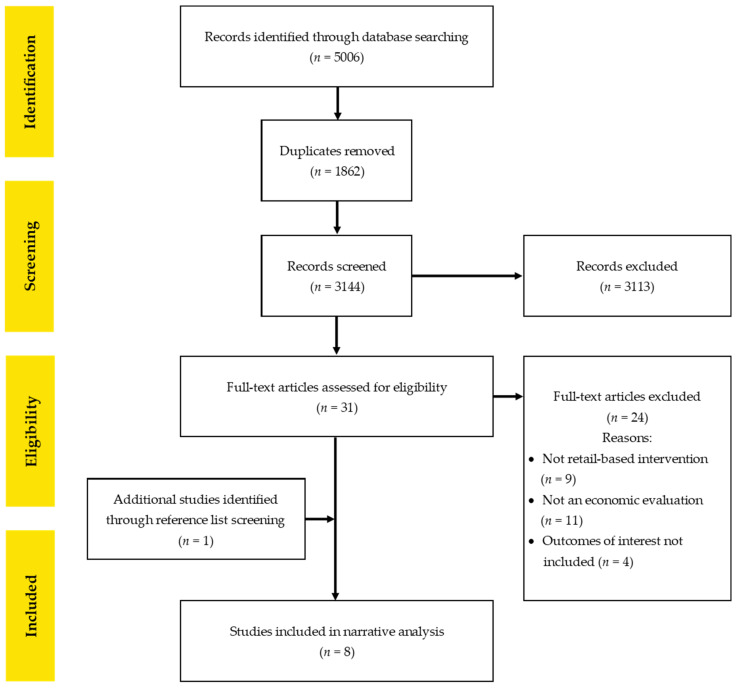
Preferred Reporting Items for Systematic Reviews and Meta-Analysis (PRISMA) flowchart summarising the inclusion and exclusion process.

A PRISMA flowchart is shown in Figure 1. The search strategy identified 5006 studies. Duplicate removal, and title and abstract screening led to 4975 studies being excluded. Full text screening was conducted on 31 studies, resulting in a further 24 studies being excluded. The remaining seven studies met the eligibility criteria and were included in the systematic review [25,26,27,28,29,30,31]. One additional study was identified by hand searching the reference lists of included studies [32].

### 3.2. Quality of Included Studies

Compliance in the reporting of the 24 items outlined in the CHEERS checklist ranged from 65% to 96% (Table S4). All eight included studies reported the target population, study perspective, and at least some of the assumptions adopted in the evaluation. Three studies reported less than 70% of the recommended items on the CHEERS checklist [27,28,30]. Three studies did not specifically state the relevant decision context [25,27,28], and three studies did not report uncertainty values for all parameters [25,28,29].

### 3.3. Intervention Characteristics

Details of the included studies are presented in Table 1 and Table 2. The eight included articles evaluated a total of 30 retail-based interventions [25,26,27,28,29,30,31,32]. The majority of the studies (*n* = 7) were published after 2015 [25,26,27,28,29,30,31]. Magnus et al. 2016 [28] modelled 12 interventions which involved providing a 20% discount on various products individually, jointly, and in combination with in-store nutrition education. Gortmaker et al. [31] modelled the cost-effectiveness of seven obesity prevention interventions, three of which met the inclusion criteria for this review. Cobiac et al. [32] modelled the cost-utility of 23 interventions to promote fruit and vegetable consumption, seven of which were in retail settings and were included in this review. The studies by Le et al. [26] and Magnus et al. 2018 [27] evaluated the cost-effectiveness of various intervention arms within a single randomised controlled trial (RCT). Three studies conducted an economic evaluation for a single intervention [25,29,30]. All studies were conducted in high-income countries [12], with the majority being undertaken in Australia (*n* = 5) [25,26,27,28,32], followed by the USA (*n* = 2) [29,31] and England (*n* = 1) [30].

When considering the 4Ps of marketing (product, price, place, and promotion), the 30 interventions in the eight included studies often included price (i.e., price discounts) (*n* = 7 [27,28]) and promotion (i.e., in-store nutrition education) (*n* = 5 [25,29,31,32]) components either on their own or in combination (*n* = 10 [26,27,28,32]). Four interventions in two studies included a product component (i.e., trans fatty acid ban and changes in nutrition standards of school meals) [30,31], and the other four interventions in one study included both product and promotion components [32]. No intervention included the place component.

The majority of the studies targeted specific groups, including the Indigenous Australian population living in remote communities [27,28], populations from socioeconomically disadvantaged backgrounds [25,29], children and adolescents [29,31], and worksite employees [32]. Four studies investigated the impact of the intervention on the general population [26,30,31,32].

All studies evaluated interventions within a single type of retail setting. Three studies evaluated interventions in supermarkets [25,26,32]. Two studies each evaluated interventions in remote community stores [27,28], schools [29,31] and restaurants [30,31]. One study evaluated interventions in fast food stores [30], and another focused on worksite cafeterias [32]. All of the interventions in supermarkets, remote community stores and worksites were from Australia [25,26,27,28,32], whilst the two school-based retail interventions were from the USA [29,31].

### 3.4. Economic Evaluation Study Characteristics by Retail Setting

#### 3.4.1. Supermarkets

In the supermarket setting, two studies conducted short-term (3 to 6 months) trial-based CEAs [25,26], and one study conducted a model-based CUA using a multi-state Markov model and conducted the evaluation over a lifetime horizon [32]. The interventions implemented at supermarkets included (1) in-store nutrition education and supermarket tours (*n* = 1) [25], and (2) combined price reductions on healthy food items and either (i) promotional materials such as lists of discounted items, flyers, or discount coupons (*n* = 2) [26,32] or (ii) skill-building materials such as newsletters and supplementary resources (*n* = 1) [26]. These interventions were evaluated either from a societal (*n* = 2) [25,26] or a health sector perspective (*n* = 1) [32].

Intervention costs and resource use in the trial-based CEAs were collected prospectively using questionnaires and sales transaction data (*n* = 2) [25,26]. The modelled study based the intervention cost components and intervention effectiveness on a RCT [32].

The results of the supermarket interventions varied from no effect to approximately A$3.29–12.70 per fruit serve increase per week [26], A$2.52 per vegetable serve increase per week [26], and A$3.39 per vegetable serve increase per day [25]. The modelled CUA showed that the intervention was not cost-effective [32].

#### 3.4.2. Remote Community Store Settings

Two model-based CUA studies, conducted by the same author group, analysed 14 interventions in remote community stores [27,28]. The interventions included healthy food price discounts alone and in combination with in-store nutrition education [27,28]. The CUAs were conducted from a societal perspective [27,28]. Magnus et al. 2016 [28] modelled the impact of the interventions based on store sales data from three remote communities, assumptions, and published data. Magnus et al. 2018 [27] modelled the long-term cost-effectiveness (using CUA) of the SHOP@RIC RCT [33] using actual intervention costs, intervention effectiveness, and the RCT sales data to estimate appropriate cross-price elasticities for the population of interest. These evaluations examined costs and benefits over a lifetime time horizon using a multi-state Markov model [27,28].

In addition to the intervention food groups (F&V, diet drinks, and water), Magnus et al. 2018 [27] examined the intervention effects on a variety of other foods, such as cereal, various types of meat products, and discretionary foods (pizza, hamburgers, snack foods and confectionary). The intervention was estimated to cost A$239,672 (price reductions) and A$433,368 (combined price reductions and in-store nutrition education), resulted in poorer health outcomes, and therefore was not cost-effective [27]. The authors reported that while the interventions had modest and lasting impacts on population F&V consumption, the measured consumption of other foods showed considerable increases in total energy and sodium intake, resulting in significant increases in BMI in the target population [27]. The modelled analyses by Magnus et al. 2016 [28] restricted the analysis to the intervention effects on the intervention food groups and found that some interventions were cost-effective. The results varied from the intervention being cost-effective at A$23,418/DALY averted (price discounts on diet drinks and water) to not cost-effective with an ICER of A$105,942/DALY (combined in-store nutrition education and price discounts on all vegetables) [28]. The difference in results from these two studies highlights that restricting the analysis of the intervention impacts to certain food groups may not reflect the intervention effect on the whole diet.

#### 3.4.3. Restaurants and Fast Food Settings

Two studies evaluated interventions in restaurant settings [30,31], with one study evaluating the same intervention (policy banning trans fatty acids) in both restaurants and fast food outlets [30]. Both evaluations were modelled policy interventions [30,31]. Gortmaker et al. [31] conducted a model-based CEA of a menu calorie labelling policy, and Allen et al. [30] conducted model-based evaluations of policies banning the use of trans-fatty acids in different settings using both CEA and CUA. These evaluations used a societal perspective [30,31]. In these two modelled evaluations, the intervention costs and resource use assumptions were based on evidence from other similar policies [30,31]. Gortmaker et al. [31] used the data from a national health survey to estimate the frequency of meals consumed outside the home. The authors then modelled the intervention effects based on a recent systematic review and meta-analysis of the impact of calorie menu labelling in restaurant settings [31]. Allen et al. [30] assumed trans fatty acid consumption in restaurants and fast food settings was proportional to food expenditure away from home [30].

To model these policy interventions, one study used an individual-level microsimulation model [31], whilst the other used a cohort model [30]. The time horizons adopted to assess costs and benefits were 5 years [30] and 10 years [31]. The modelled CEA of the menu calorie labelling at restaurants resulted in a mean ICER of A$20.55 (95%CI: -A$192.52; A$242.47) per BMI unit reduced [31]. The Allen et al. [30] results were disaggregated to incremental health outcomes and various cost components. The study concluded that the intervention in both restaurant and fast food settings were dominant (health promoting and cost-saving) [30].

#### 3.4.4. Cafeteria Settings and Vending Machines

##### School Cafeterias

Two studies evaluated three interventions in school cafeterias using a CEA framework, one trial-based analysis over a 5-week time horizon [29] and one model-based study using a 10-year time horizon [31]. Gortmaker et al. [31] included two interventions: nutrition standards for school meals provided by schools and nutrition standards for all foods and beverages sold in school settings. Ladapo et al. [29] evaluated an intervention comprising various changes to the school food environment including the school cafeteria, providing free taste tests for healthy foods, school-wide multimedia marketing to encourage heathy eating and physical activity, and the installation of a filtered water system [29].

The evaluations in these two studies were undertaken from different perspectives (societal [31] and school perspectives [29]). The study by Ladapo et al. [29] did not consider the opportunity cost of teachers’ time for their involvement in the intervention nor cafeteria staff time costs, the reasoning being that teachers were already employed by schools and food preparation activities of the cafeteria staff were part of their normal duties [29].

The intervention effectiveness for the Ladapo et al. [29] study was based on the Students for Nutrition and Exercise (SNaX) RCT. Gortmaker et al. [31] based the intervention effects on one natural experiment cross-sectional study analysing the associations between state laws regulating school meal nutrition content and student weight status, and one retrospective cohort study examining the impacts of state laws around snacks sold in schools on BMI status. The effectiveness and cost-effectiveness of the interventions implemented at school settings varied from no intervention effect on the number of portions of vegetables served during meals [29] to A$1.88 per additional portion of fruit served during meals [29], A$2.65 per reduced unit of snacks sold (school environment change intervention) [29], A$9.58 per BMI unit reduced (nutrition standards for all foods and beverages sold in schools) to A$83.22 per BMI unit reduced (nutrition standards for school meals) [31].

##### Worksite Cafeterias and Vending Machines

Cobiac et al. [32] modelled various interventions at worksite cafeterias and vending machines from a health sector perspective. Each modelled intervention, including intervention components, cost components and intervention effects, was based on a published study undertaken at worksites [32]. Cobiac et al. [32] conducted model-based CUAs using multi-state Markov modelling over a lifetime time horizon [32]. The retail interventions included (i) menu labelling, (ii) in-store nutrition education, and (iii) provision of promotional materials and skill-building (i.e., pamphlets and brochures) and changes in food policies for the whole organisation [32]. The cost components and the source of the data were not well described, and the authors noted that there was substantial uncertainty around the intervention effect [32]. ICER results indicated that only one intervention in worksite cafeterias that included the display of information sheets near food products (i.e., caloric value of food translated to number of minutes to perform occupational activity) in cafeterias and vending machines was potentially cost-effective, with an ICER of A$72,638/DALY averted. The modelled intervention was estimated to increase F&V consumption by 2.5 serves per person per day [32]. The cost-effective intervention had a lower cost per participant (A$170.00) compared to other interventions [32].

### 3.5. Key Assumptions Used in the Economic Evaluations

**Figure 2:**
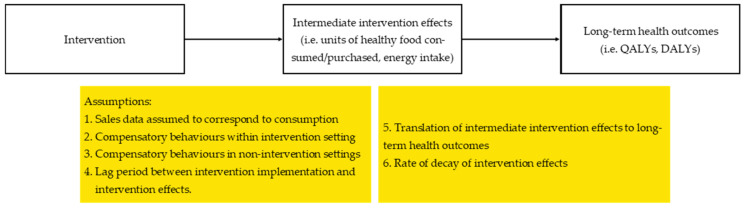
Intervention effect pathway and assumptions. Notes: DALYs: Disability-adjusted life years, QALYs: Quality-adjusted life years.

Several assumptions were required to estimate the intermediate intervention effects and long-term health impacts of retail interventions. The logic pathway of intervention effects and the assumptions reported or required are shown in Figure 2. Table 3 documents the assumptions reported in each study. In retail intervention evaluations, assumptions around how intermediate intervention effects such as change in consumption impact longer term health outcomes are required. The cost-effectiveness results from within trial evaluations required fewer assumptions as results were reported as the cost per change in intermediate intervention effects. However, these studies were also unable to draw conclusions on the long-term cost-effectiveness of interventions.

#### 3.5.1. Sales Data Assumed to Correspond to Consumption

The measurement of the impact of the intervention on consumption varied across studies. Two studies measured consumption both directly using validated self-report surveys pre and post intervention and indirectly using sales data [25,26]. One study used only sales data to estimate intervention impacts on the consumption of various intervention and non-intervention foods [27]. A key assumption required when using self-reported consumption or sales data is that any change in consumption is exclusively attributable to the intervention under study. This was a reasonable assumption in the two RCT studies [25,26] as influences on consumption other than the intervention could be assumed to be similar across both the intervention and control arms. Interestingly, in the SHELF study [26], there was no statistically significant effect on self-reported consumption data, despite sales data showing that participants purchased significantly more intervention foods. This contrasts to the Ball et al. [25] study, which reported that the intervention led to a significant increase in self-reported vegetable consumption, even though there was no statistically significant difference in vegetable purchases based on sales data. This illustrates the inconsistency between food consumption measured by self-report methods versus sales data.

Sales data were the most often used measure of intervention effectiveness and were used as a proxy for changes in consumption. There were different ways in which sales data were collected, including customer loyalty cards [25,26] and store sales data [27]. The key assumption required when using sales data as a proxy for consumption is that food purchase patterns of the target population at other stores remain unchanged or that the intervention store is the major food source of the target population. These assumptions were not required in the study by Magnus et al. 2018 [27], given that this was an RCT and the intervention store accounted for 96% of foods available in the remote community.

Modelled studies estimated intervention effectiveness based on the literature, such as systematic reviews and meta-analyses of relevant retail interventions [31], trials of retail interventions [31,32], a retrospective cohort study examining the impacts of state laws on snacks sold in schools [31], or published food price elasticities [28]. In some cases where effect size data were not available, assumptions were required to estimate the proportion of individual consumption that would change due to the intervention in any specific retail setting, which could then be used as a proxy to predict intervention impacts on overall consumption. One study drew on national food expenditure data to estimate the proportion of food consumed away from home to gauge the intervention effect size [30]. Given that effect sizes were derived indirectly from other studies, the validity and reliability of these intervention effects depended on the quality of the original studies.

#### 3.5.2. Compensatory Behaviours Within Intervention Settings and in Non-Intervention Settings

Compensatory behaviours relate to the purchasing and consumption of food and beverages that are not targeted by the intervention or outside the intervention setting. Four of the eight included studies stated assumptions around compensatory behaviours of the target population [26,27,28,31]. Two studies used sales of not only intervention targeted food items, but all purchases within the store [26,27] to assess within-store compensatory purchases. Given that the intervention stores in the Magnus et al. [27] study were the major food source of the remote Indigenous population, by collecting sales data of both intervention and non-intervention foods and beverages, this study was able to examine the intervention impacts on the whole diet of the population [27]. One study explicitly stated that compensatory purchasing at non-intervention stores was not considered in the analysis [31].

Magnus et al. 2018 [27] reported that a decrease in the price of F&V was associated with an increase in the purchase of both F&V and other non-intervention foods, such as cakes, biscuits, and ready-to-eat foods, which resulted in increased modelled BMI, and therefore the intervention was not cost-effective [27]. The contrasting results in the Magnus et al. 2016 [28] study, which used published price elasticities to calculate the intervention impacts on intervention food items only, demonstrated that the consideration of intervention impacts on whole diets is instrumental in accurately estimating the cost-effectiveness of retail interventions.

In the restaurant, cafeteria, and fast food settings, it is not clear whether assumptions related to compensatory behaviours are more or less important, given that the consumption of the targeted food represents a smaller proportion of the total diet. Retail interventions in these settings did not consider compensatory purchasing at non-intervention locations [31].

One study evaluating a restaurant intervention assumed that reductions in calories ordered or purchased would lead to equivalent reductions in consumption, without any consideration of food wastage [31]. Such an assumption is problematic, given evidence from an intervention in the school settings which showed that healthier meals resulted in an increased amount of food plate wastage [34,35]. None of the interventions in supermarkets or remote stores considered food wastage when estimating the effectiveness and cost-effectiveness of a retail intervention to increase the purchase and consumption of healthy foods.

#### 3.5.3. Lag Period Between Intervention Implementation and Intervention Effects

Unlike trial-based evaluations where the impact of an intervention on primary intermediate intervention effects were measured at specific time points, in the model-based evaluations, the timing of intervention impacts was largely unspecified. One model-based study assumed that the intervention took around 18 to 36 months to have an impact on BMI [30]. The other studies implicitly assumed that the intervention impact was immediate [27,28,30,32].

#### 3.5.4. Translation of Intermediate Intervention Effects to Long-Term Health Outcomes

Four studies that modelled the intermediate intervention effects to long-term DALYs or quality-adjusted life years (QALYs) employed different logic pathways [27,28,30,32]. Amongst these studies, the risk factors impacted by the interventions included sodium intake and BMI (*n* = 2) [27,28], trans-fatty acid (*n* = 1) [30] and F&V consumption (*n* = 1) [32]. The diseases attributable to each of these risk factors were different, although some diseases are caused by more than one risk factor, and vice versa. The most commonly included diseases were heart disease (*n* = 4) [27,28,30,32], various types of cancer (*n* = 3) [27,28,32], and type 2 diabetes (*n* = 2) [27,28]. Allen et al. [30] only considered intervention impacts on one disease to estimate QALYs, while the other studies included several obesity-related diseases to estimate DALYs [27,28,32]. In Magnus et al. 2016, each risk factor (F&V consumption, sodium intake, and BMI) was assumed to impact the included diseases [27], whilst in the Cobiac et al. [32] study only one risk factor (F&V consumption) was used to estimate changes in disease epidemiology.

#### 3.5.5. Rate of Decay of Intervention Effects

In the modelling of long-term health impacts, the assumption about the maintenance of the intervention effect is pivotal. One study explicitly stated that limited evidence on the sustainability of behavioural changes is the key unknown variable in modelled economic evaluations of preventive interventions [32]. Two of the five model-based evaluations [28,32] included assumptions around the maintenance of intervention effects. Cobiac et al. [32] and Magnus et al. 2016 [28] assumed intervention effects would decay 50% annually based on the pattern of weight regain from weight loss programmes; this may not accurately estimate the sustainability of the effects of retail interventions. The modelled CUA reported in Magnus et al. 2018 [27] assumed a 50% decay of intervention impacts annually and justified the decision using an analysis of the trial data and references to other published studies. Gortmaker et al. [31] assumed that the intervention would be ongoing and full intervention effects would be maintained throughout the 10-year time horizon of the evaluation.

In the majority of trial-based studies, intervention effects were measured directly after a specified period of intervention implementation (ranging from 3 to 6 months) and at 6-months follow up; therefore, the short term sustainability of intervention effects post intervention completion were more accurately measured and incorporated into the evaluations [25,26]. While Ball et al. [25] observed small intervention impacts at 6 months post-intervention, Le et al. [26] reported a deceased intervention effect at the same time point.

## 4. Discussion

Interventions in retail settings can influence healthy food purchasing and consumption behaviours and therefore impact health outcomes [14]. Whilst the economic credentials of these retail interventions are important to consider when deciding which interventions to invest in, this review only identified eight studies that conducted economic evaluations of healthy food retail interventions [25,26,27,28,29,30,31,32]. Future healthy food-retail intervention studies should incorporate an economic evaluation in order to build this evidence base.

The key finding of this review was that the cost-effectiveness of food retail interventions varied. Many studies evaluated interventions using a CEA framework reporting ICERs for an array of health outcomes. The ultimate assessment of the value for money of these interventions is left to the decision-maker, as there are no willingness to pay thresholds facilitating conclusions to be drawn on the cost-effectiveness of these interventions. In supermarket settings, the only study that reported CUAs indicated that the intervention that included a A$0.77 coupon to redeem on F&V and in-store promotion was not cost-effective [32]. In remote community store settings, the evaluation based on trial data which included an estimate of compensatory purchasing of non-target food groups showed that the interventions, which included price reductions on F&V, diet drink, and water and a combination of price reductions and in-store nutrition education, were not cost-effective [27], despite increasing the purchasing of targeted foods. In restaurant and fast food store settings, a modelled policy banning trans fatty acids was assessed as dominant [30]. In the school cafeteria setting, none of the studies reported long-term health outcomes expressed as ICERs, meaning it is not possible to draw conclusions on the long-term cost-effectiveness of these retail interventions [29,31]. In worksite cafeteria settings, most of the interventions evaluated in the study by Cobiac et al. [32] were not cost-effective. It should be noted that all included studies were conducted in high-income countries [12], and therefore it is unclear whether these findings are generalisable to middle- or low-income countries where food-retail settings, food purchasing, and consumption patterns may vary.

Intervention costing was performed alongside a trial either prospectively or retrospectively based on published data. Prospective costing alongside a trial can provide more accurate estimates of intervention costs compared to modelled analyses that used published literature to estimate the costs of implementation in the relevant country setting. Various costing perspectives were used, and therefore the cost items included in the analyses also varied. Only one study included impacts on retailer profits [30]; however, the importance of considering business implications for the retailer has been recently highlighted [36].

The assumptions used in the economic evaluations were generally not based on good quality evidence or data. Assumptions about compensatory behaviours were missing or poorly measured in the majority of studies. This issue should be addressed in future studies given its likely impact on the effectiveness and therefore the cost-effectiveness of healthy food retail interventions. Innovative technologies that can capture complete dietary intake and allow accurate estimations of the type of food consumed, serving size, nutrition profile, and the time of consumption will be useful in filling this data gap. Advancements in measurement could include the use of handheld scanners or smartphones to capture Universal Product Codes and Global Trade Item codes and wearable sensors that automatically record food consumption via hand-to-mouth movement or swallowing sensors [37]. Whilst these new technologies could aid measurement of compensatory behaviours, their validity has not yet been systematically evaluated [1,37].

The rate of decay of intervention effects is likely to be a key driver of cost-effectiveness results, especially in model-based studies. It is unknown whether intervention duration affects the sustainability of intervention impacts. Future research should also examine the relationship between intervention type, setting, duration, and the sustainability of intervention effects.

The studies by Magnus et al. 2018 [27] and Magnus et al. 2016 [28] highlight the issues related to limiting the analysis of sales data to intervention-related food and drink items, especially when the intervention has a price component. However, the authors highlighted that although the cost-effectiveness results were not favourable, the intervention, which resulted in increased F&V and other food purchases, was likely to be beneficial to the remote community, since the population might be undernourished and their baseline dietary energy intake was below the recommended daily energy requirements (8700 kilojoules) [27].

There was evidence that participants purchased foods from various food outlets [25,26], which highlights the importance of capturing all food purchases rather than those in intervention stores alone. This may be particularly important for certain types of interventions; for example, when the intervention restricts availability or price of unhealthy foods, participants may compensate by purchasing unhealthy foods at non-intervention retail settings [38]. Although some studies ask participants to list the major food stores where they purchase food [39,40,41], a more comprehensive approach to assessing compensatory purchases at non-intervention retail settings is required.

Sales data were used to estimate intervention effects, with data collected either using customer loyalty cards [25,26] or store sales data [27]. Whilst the first method enables the measurement of household-level food and beverage purchase volume, it has several issues, including the compliance with the use of the card and the inappropriate use of the card to purchase discounted foods for non-household members [25,26]. Store sales data allow for the capture of all sales in the intervention stores but lacks the ability to examine the individual level purchase data and the required assumption that the population the stores are servicing remains constant. Another limitation is that store sales data do not allow for the assessment of any heterogeneity of the impact on population subgroups [27].

While the analysis of sales data is considered an accurate, inexpensive and objective method of examining population food purchasing behaviours [42,43,44], this should be used, where possible, in conjunction with other methods to measure and validate retail intervention effects on consumption. The amount of food purchased does not always equate to the amount of food consumed by a household [45,46,47]. Households are identified as a major contributor to food waste [46,48]. For example, fresh foods such as F&V are often more perishable and at higher risk of wastage [46,49], and therefore interventions that aim to increase F&V consumption may overestimate the impact of the intervention if wastage is not measured. Other limitations of sales data include the difficulty in accessing these data from retailers due to confidentiality, variations in data quality from different retailers (i.e., lack of product description or information about weight of food product sold), lack of transparency on data-collection methods, and the sometimes high cost of purchasing commercial sales data [43]. Despite its limitations, sales data provide a level of objectiveness of purchase data that is representative of specific geographic locations [42,43]. It is suggested that sales data can provide more granular information compared to dietary survey data and can be updated frequently, thereby helping researchers to more readily measure and monitor food purchase patterns of the population [43]. Another advantage is that sales data, especially from commercial companies, can aid multinational comparisons of population food purchasing patterns and lower participant burden [43,44]. Regardless of the method used to capture food consumption, it is imperative to note that there may be reasons for changes in consumption that are unrelated to the intervention, such as seasonal changes [38,50]. These impacts can be accounted for by using an RCT study design.

Two studies collected self-reported food intake alongside sales data [25,26]. Although self-reported dietary data can potentially provide individual-level consumption data, it is unknown whether self-reported data are a good representation of actual consumption. The New Zealand Adult Nutrition Survey reports that self-reported data suffer from systematic underreporting and advises users to take extra caution when interpreting energy intake data measured using the dietary recall method [51]. Another study found that people often reported higher intake of healthier food and lower intake of unhealthy food than their actual purchases [52].

The key strength of this review was the extensive and rigorous search strategy, covering 14 databases, and the inclusion of all food retail settings. The review therefore comprehensively captured all economic evaluations of health-promoting food retail interventions. There were also some important limitations. Firstly, assumptions related to the intervention logic pathway for the included studies were limited to the details reported in the published articles. Given the limitations in article length for various journals, the full details of the assumptions may not have been reported in the published articles. Contacting the authors for additional information might have provided a better understanding of the modelling assumptions. Secondly, the methodological quality of the included studies was assessed using the CHEERS checklist, which is designed to evaluate the reporting quality of economic evaluations rather than the methodological quality of studies [21]. Various checklists, such as the British Medical Journal checklist [53], the Consensus on Health Economic Criteria (CHEC)-list [54], the Philips checklist [55], and the CHEERS checklist [21], have been used to assess the quality of economic evaluations [56]. Common issues raised by reviewers when using these checklists include: (i) the checklist is subjective in its nature, (ii) the scores are not representative of the quality of evaluations, (iii) the checklists are not sufficiently comprehensive, and (iv) the use of these checklists does not affect the conclusion of the review of economic evaluations [56]. The future development of a methodological quality assessment checklist for economic evaluation studies may help to reach more accurate conclusions about the economic credentials of interventions, especially when published studies report mixed results. The checklist should place more emphasis on the rationale for the assumptions adopted in economic evaluations, the supporting data for these assumptions, and how the assumptions consequentially affect the cost-effectiveness results. Finally, some relevant studies may have been missed, as this review was limited to studies published in English.

## 5. Conclusions

This review is the first to synthesise evidence of the cost-effectiveness of health promoting interventions in food retail settings. It also examined the assumptions adopted in these economic evaluations. The review found that the economic evidence of healthy food retail interventions is limited and restricted to four food retail settings. The cost-effectiveness results of interventions in each of these settings were varied and inconclusive. The studies adopted a range of assumptions to inform evaluations; however, some key assumptions were missing or poorly reported in the studies, with the impact of compensatory behaviours likely to be the most influential on cost-effectiveness results.

## Figures and Tables

**Table 1:** Characteristics of included economic evaluations.

Author	Country, Target Population	Evaluation Type, Study Design, Perspective, Time Horizon	Reference Year, Discount Rate, Currency	Intervention and Comparator
**Supermarkets**				
Ball et al. 2016 [25]	Australia, socio-economically disadvantaged female shoppers	CEA, within-trial evaluation, societal perspective, 6 month intervention, 12 month follow up (6 months post intervention)	2012, NA, A$	I: Behaviour change intervention (education and skill-building materials) C: Status quo
Le et al. 2016 [26]	Australia, female shoppers	CEA, within-trial evaluation, societal perspective, 3 months	2012, NA, A$	I1: Skill-building (non-retail setting) I2: 20% price reduction on F&V, water and diet or low-calorie beverages at checkouts; and in-store promotion I3: I1 and I2 C: Status quo
Cobiac et al. [32]	Australia, supermarket shoppers	CUA, modelled, health sector perspective, lifetime	2003, 3%, A$	I1: A$0.77 coupon to redeem on F&V, and in-store promotion C: Status quo
**Remote Community Stores**				
Magnus et al. 2016 [28]	Australia, 2011 Australian Indigenous population	CUA, modelled, societal perspective, life-time	2011, 3%, A$	20% price reductions for: I1: All fruit I2: Fresh vegetables only I3: All vegetables I4: All F&V I5: Diet drinks and water I6: All F&V, diet drinks and water In-store nutrition education and 20% price reductions for: I7: All fruit I8: Fresh vegetables only I9: All vegetables I10: All F&V I11: Diet drinks and water I12: All F&V, diet drinks and water C: Status quo
Magnus et al. 2018 [27]	Australia, population living in remote Indigenous communities in Northern Australia	CUA, modelled from trial data, partial societal perspective (including health and retail sector impacts), life-time	2011, 3%, A$	I1: 20% price reduction for F&V, diet drink and water for 24 weeks I2: I1 in combination with in-store nutrition education for 24 weeks C: Status quo
**Restaurants and Fast Food Stores**				
Gortmaker et al. 2015 [31]	USA, general population	CEA, modelled, societal perspective, 10 years	2014, 3%, USD	I1: Menu calorie labelling in restaurants C: Status quo
Allen et al. 2015 [30]	England, adults >25 years	CEA, modelled, societal perspective, 5 years	2015, 3.5%, GBP	I1: Ban on trans fatty acids in restaurants I2: Ban on trans fatty acids in fast food outlets C: Status quo
		CUA, modelled, societal perspective, 5 years		
**School Cafeterias**				
Gortmaker et al. 2015 [31]	USA, school children from kindergarten to grade 12	CEA, modelled, societal perspective, 10 years	2014, 3%, USD	I2: Nutrition standards for school meals I3: Nutrition standards for all foods and beverages sold in schools C: Status quo
Ladapo et al. 2016 [29]	USA, low-income grade 6–8 students	CEA, within-trial evaluation, school perspective, 5 weeks	2014, NA, USD	I1: School-wide environmental changes to promote water and healthy foods consumption; and physical activity (retail intervention included preparation of healthier food taste tests in cafeterias, other interventions included a peer leader club and school-wide multimedia marketing) C: Status quo
**Worksite Cafeterias and Vending Machines**				
Cobiac et al. 2010 [32]	Australia, worksite employees, cafeterias	CUA, modelled, health sector perspective, lifetime	2003, 3%, A$	I2-5: Each modelled intervention was based on a single published study. Each intervention included some or all of the following components: menu labelling, in-store nutrition education, changes to catering food policies and food labelling in cafeterias and vending machines I6: Food demonstration in cafeterias, food labelling, special events (e.g., vegetable soup day), and provision of skill building materials (e.g., pamphlets and brochures) I7: Display of information sheets near food products (e.g., caloric value of food translated to number of minutes to perform occupational activity) in cafeterias and vending machines C: Status quo

Notes: A$: Australian dollar; BMI: body mass index; C: comparator; CEA: cost-effectiveness analysis; CI: confidence interval; CUA: cost-utility analysis; F&V: fruit and vegetable; GBP: British pound sterling; I: intervention; NA: not applicable; USA: United States of America; USD: American dollars.

**Table 2:** Intervention cost components, outcomes, and cost-effectiveness results.

Author	Intervention Cost(s)	Intermediate Intervention Effects(s)	Outcome(s) of Interest	Incremental Cost-Effectiveness Ratio (A$2020)
**Supermarkets**				
Ball et al. 2016 [25]	Intervention materials Staff time including overheads Participant time Purchase of intervention food products Participant travel expenses	NA	(1) F&V purchases (2) F&V self-reported intake	No effect on fruit intake $3.39 (95%CI: NR) per increased serve of vegetables consumed per participant per day
Le et al. 2016 [26]	I2 and I3: Staff time including overhead Participant time Purchase of intervention food products Participant travel expenses Intervention materials	NA	(1) F&V purchases (2) F&V self-reported intake (3) Low-calorie carbonated diet beverages, and water purchases (4) Low-calorie carbonated diet beverages, and water self-reported intake	I1: No difference in all primary outcomes compared to comparator I2: $2.52 (95%CI: NR) per increased serve of vegetables purchased per week $3.29 (95%CI: NR) per increased serve of fruit purchased per week No difference in beverage purchases and intake. No difference in vegetable intake. I3: $12.70 (95%CI: NR) per increased serve of fruit purchased per week. No difference in vegetable and beverage purchases No difference in fruit, vegetable, and beverage intake
Cobiac et al. 2010 [32]	I1: Intervention materials Monetary incentives	Modelled F&V intake	Modelled DALYs averted	I1: $3,863,748 (95%CI: NR) per DALY averted The intervention resulted in 0.030 (95%CI: −0.34; 0.40) increase in serves of F&V per day (not statistically significant)
**Remote community stores**				
Magnus et al. 2016 [28]	I1–6: Price discount Staff time Intervention materials I7–12: Price discount Staff time Intervention materials Participant time Participant travel expenses	Modelled sodium intake, total energy intake and BMI	Modelled DALYs averted	I1: $30,110 (95%CI: $18,958; $44,607) per DALY averted I2: $37,916 (95%CI: $22,304; $56,874) per DALY averted I3: $76,947 (95%CI: $55,759; $101,481) per DALY averted I4: $49,067 (95%CI: $36,801; $64,680) per DALY averted I5: $23,418 (95%CI: dominated *; $535,285) per DALY averted I6: $40,146 (95%CI: dominated *; $356,857 per DALY averted I7: $53,529 (95%CI: $40,146; $70,256) per DALY averted I8: $68,026 (95%CI: $50,183; $88,099) per DALY averted I9: $105,942 (95%CI: $82,523; $133,821) per DALY averted I10: $56,874 (95%CI: $44,607; $72,487) per DALY averted I11: $37,916 (95%CI: dominated *; $791,776) per DALY averted I12: $42,377 (95%CI: dominated *; $390,312) per DALY averted
Magnus et al. 2018 [27]	I1: Price discount Staff time Staff travel expenses Intervention materials I2: Price discount Staff time Staff travel expenses Intervention materials Participant time	(1) Store sales of F&V, water and artificially sweetened soft drinks (2) Total weight, energy and sodium of food purchases (3) Modelled BMI	Modelled DALYs averted	I1 and 2: Increased purchase of F&V and other non-discounted foods resulting in modelled increase in BMI of 2.38 (95%CI: 0.81; 4.62) (I1) or 2.37 (95%CI: 0.78; 4.75) (I2). During the discount period, the negative impact on DALYs averted was from −21 (95%CI: −28; −15) to −36 (95%CI: −47; −25). At follow−up, the negative impact on DALY averted was from −48 (95%CI: −60; −36) to −45 (95%CI: −58; −34). Incremental intervention costs: I1: $239,672 (95%CI: NR) I2: $433,368 (95%CI: NR) Interventions were not cost-effective
**Restaurants and fast food stores**				
Gortmaker et al. 2015 [31]	I1: Staff time Nutrition database accessing fee Compliance monitoring	Modelled calorie intake	Modelled BMI	I1: $20.55 (95%CI: −$192.52; $242.47) per BMI unit reduced
Allen et al. 2015 [30]	Legislation Compliance monitoring Product reformulation Industry loss profitability	Modelled trans fatty acid intake	Modelled deaths from coronary heart disease prevented or postponed	I1: 1800 (95%CI: 700; 3400) deaths from coronary heart disease averted or 0.7% reduction. Total annual costs: $185.44M (95%CI: NR) Net costs saving (excluding reformulation cost): $109.87M (95%CI: $215.57M; $6.03M) Net costs saving (including reformulation cost): $0.00M (95%CI: $105.47M; −$103.85M) I2: 2600 (95%CI: 1200; 4600) deaths from coronary heart disease averted or 1.0% reduction Total annual costs: $220.67M (95%CI: NR) Net costs saving (excluding reformulation cost): $174.08M (95%CI: $316.41M; $34.31M) Net costs saving (including reformulation cost): $28.98M (95%CI: $171.30M; −$110.80M)
			Modelled QALYs gained	I1: Dominant ^#^ QALY gained: 2100 (95%CI: 700; 3900) Healthcare cost savings: $26.19M (95%CI: $11.59M; $41.26M) Averted productivity loss: $36.62M (95%CI: $15.99M; $57.49M) Informal care savings: $122.62M (95%CI: $54.01M; $192.39M) I2: Dominant ^#^ QALY gained: 3000 (95%CI: 1100; 5200) Healthcare cost savings: $35.23M (95%CI: $15.53M; $55.17M) Averted productivity loss: $50.53M (95%CI: $22.25M; $79.28M) Informal care savings: $164.11M (95%CI: $72.09M; $257.53M)
**School cafeterias**				
Gortmaker et al. 2015 [31]	I2: State and local government: Reimbursements for meals Kitchen equipment for schools Compliance monitoring School costs: Meal Staff time I3: School costs: Staff time to keep records of compliance Training	Modelled calorie intake	Modelled BMI	I2: $83.22 (95%CI: −$209.49; $292.06) per BMI unit reduced I3: $9.58 (95%CI: $3.67; $12.22) per BMI unit reduced
Ladapo et al. 2016 [29]	Peer leader activities School-wide multimedia marketing School food environment changes	(1) Portions of F&V served (2) Number of snacks sold	(1) Portions of F&V served (2) Number of snacks sold	(1) No intervention effect on portions of vegetables served $1.88 (95%CI: NR) per additional portion of fruit served during meals (2) $2.65 (95%CI: NR) per reduced unit of snacks sold
**Worksite cafeterias and vending machines**				
Cobiac et al. 2010 [32]	I2: Workshop Nutrition displays Cafeteria promotion Advisory board Time I3–5: Workshop Nutrition displays Cafeteria promotion Advisory board Time Non-tailored documents I6: Workshop Nutrition displays Cafeteria promotion Advisory board Time Family involvement I7: Nutrition displays Cafeteria promotion Non-tailored documents	Modelled F&V intake	DALYs averted	I2: $11,436,695 (95%CI: NR) per DALY averted I3: $1,220,945 (95%CI: NR) per DALY averted I4: $494,560 (95%CI: NR) per DALY averted I5: $1,854,599 (95%CI: NR) per DALY averted I6: $664,565 (95%CI: NR) per DALY averted I7: $72,639 (95%CI: NR) per DALY averted (50% probability of being cost-effective)

Notes: ^#^ intervention resulted in more health benefits and less cost compared to the comparator; * intervention resulted in less health benefits and more cost compared to the comparator; A$: Australian dollar; BMI: body mass index; CI: confidence interval; DALYs: disability-adjusted life years; F&V: fruit and vegetable; GBP: British pound sterling; I: intervention; NR: not reported; QALYs: quality-adjusted life years.

**Table 3:** Assumptions reported in economic evaluations.

Studies	Trial-Based Evaluations			Model-Based Evaluations				
	CEA			CEA & CUA		CUA		
Assumption	Ball et al. [25]	Ladapo et al. [29]	Le et al. [26]	Gormaker et al. [31]	Allen et al. [30]	Cobiac et al. [32]	Magnus et al. 2016 [28]	Magnus et al. 2018 [27]
Sales data assumed to correspond to consumption	NA	No	NA	Yes	No	No	No	No
Compensatory behaviours within intervention setting	No	No	YE	Yes	No	No	Yes	YE
Compensatory behaviours in non-intervention settings	No	No	No	Yes	No	No	YE	YE
Lag period between intervention implementation and intervention effects	NA	NA	NA	YE	No	No	No	No
Translation of intermediate intervention effects to long-term health outcomes	NA	NA	NA	No	YE	YE	YE	YE
Rate of decay of intervention effects	NA	NA	NA	YE	No	YE	Yes	YE

Notes: NA not applicable; No: assumption was not reported; Yes: some assumptions were reported; YE: assumptions were supported by evidence; CEA: cost-effectiveness analysis; CUA: cost-utility analysis.

## Data Availability

The data presented in this study are available within this article and the Supplementary Materials.

## Supplementary Materials

The following are available online at https://www.mdpi.com/1660-4601/18/3/1356/s1, Table S1: PRISMA checklist, Table S2: List of search terms used for the database searches, Table S3: List of Medical Subject Headings terms used for the database searches, Table S4: CHEERs Checklist of included studies.

## References

[1] Afshin A., Sur P.J., Fay K.A., Cornaby L., Ferrara G., Salama J.S., Mullany E.C., Abate K.H., Abbafati C., Abebe Z. (2019). Health effects of dietary risks in 195 countries, 1990–2017: A systematic analysis for the Global Burden of Disease Study 2017. Lancet.

[2] World Health Organization Noncommunicable Diseases Geneva, Switzerland: World Health Organization, Regional Office for the Eastern Mediterranean. http://www.emro.who.int/noncommunicable-diseases/causes/unhealthy-diets.html.

[3] World Health Organization Global Health Observatory (GHO) Data Geneva, Switzerland: World Health Organization. https://www.who.int/gho/ncd/risk_factors/unhealthy_diet_text/en/.

[4] Norat T., Chan D., Lau R., Aune D., Vieira R., Corpet D. (2010). The associations between food, nutrition and physical activity and the risk of colorectal cancer. WCRF/AICR Systematic Literature Review Continuous Update Project Report.

[5] World Health Organization (2003). Diet, Nutrition, and the Prevention of Chronic Diseases: Report of a Joint WHO/FAO Expert Consultation.

[6] Swinburn B.A., Sacks G., Hall K.D., McPherson K., Finegood D.T., Moodie M.L., Gortmaker S.L. (2011). The global obesity pandemic: Shaped by global drivers and local environments. Lancet.

[7] Needham C., Orellana L., Allender S., Sacks G., Blake M.R., Strugnell C. (2020). Food retail environments in Greater Melbourne 2008–2016: Longitudinal analysis of intra-city variation in density and healthiness of food outlets. Int. J. Environ. Res. Public Health.

[8] Moayyed H., Kelly B., Feng X., Flood V. (2017). Is living near healthier food stores associated with better food intake in regional Australia?. Int. J. Environ. Res. Public Health.

[9] Miller L.J., Joyce S., Carter S., Yun G. (2014). Associations between childhood obesity and the availability of food outlets in the local environment: A retrospective cross-sectional study. Am. J. Health Promot..

[10] Paquet C., Coffee N.T., Haren M.T., Howard N.J., Adams R.J., Taylor A.W., Daniel M. (2014). Food environment, walkability, and public open spaces are associated with incident development of cardio-metabolic risk factors in a biomedical cohort. Health Place.

[11] Adam A., Jensen J.D. (2016). What is the effectiveness of obesity related interventions at retail grocery stores and supermarkets?—A systematic review. BMC Public Health.

[12] The World Bank High Income. https://data.worldbank.org/country/XD.

[13] Cameron A.J., Charlton E., Ngan W.W., Sacks G. (2016). A systematic review of the effectiveness of supermarket-based interventions involving product, promotion, or place on the healthiness of consumer purchases. Curr. Nutr. Rep..

[14] Hartmann-Boyce J., Bianchi F., Piernas C., Riches S.P., Frie K., Nourse R., Jebb S.A. (2018). Grocery store interventions to change food purchasing behaviors: A systematic review of randomized controlled trials. Am. J. Clin. Nutr..

[15] Mah C.L., Luongo G., Hasdell R., Taylor N.G.A., Lo B.K. (2019). A Systematic Review of the Effect of Retail Food Environment Interventions on Diet and Health with a Focus on the Enabling Role of Public Policies. Curr. Nutr. Rep..

[16] Karpyn A., McCallops K., Wolgast H., Glanz K. (2020). Improving consumption and purchases of healthier foods in retail environments: A systematic review. Int. J. Environ. Res. Public Health.

[17] Ananthapavan J., Sacks G., Moodie M., Carter R. (2014). Economics of obesity—Learning from the past to contribute to a better future. Int. J. Environ. Res. Public Health.

[18] Moher D., Liberati A., Tetzlaff J., Altman D.G. (2010). Preferred reporting items for systematic reviews and meta-analyses: The PRISMA statement. Int. J. Surg..

[19] Hupe M. (2019). EndNote X9. J. Electron. Resour. Med. Libr..

[20] Veritas Health Innovation (2020). Covidence systematic review software Melbourne, Australia: Veritas Health Innovation. www.covidence.org.

[21] Husereau D., Drummond M., Petrou S., Carswell C., Moher D., Greenberg D., Augustovski F., Briggs A.H., Mauskopf J., Loder E. (2013). Consolidated health economic evaluation reporting standards (CHEERS) statement. Int. J. Technol. Assess. Health Care.

[22] OECD (2017). Purchasing power parities (PPP): OECD. https://www.oecdilibrary.org/content/data/1290ee5a-en.

[23] Australian Institute of Health Welfare (2019). Health Expenditure Australia 2017–18.

[24] Australian Institute of Health Welfare (2009). Health Expenditure Australia 2007–08.

[25] Ball K., McNaughton S.A., Le H.N.D., Abbott G., Stephens L.D., Crawford D.A. (2016). ShopSmart 4 Health: Results of a randomized controlled trial of a behavioral intervention promoting fruit and vegetable consumption among socioeconomically disadvantaged women. Am. J. Clin. Nutr..

[26] Le H.N.D., Gold L., Abbott G., Crawford D., McNaughton S.A., Mhurchu C.N., Pollard C., Ball K. (2016). Economic evaluation of price discounts and skill-building strategies on purchase and consumption of healthy food and beverages: The SHELf randomized controlled trial. Social Sci. Med..

[27] Magnus A., Cobiac L., Brimblecombe J., Chatfield M., Gunther A., Ferguson M., Moodie M. (2018). The cost-effectiveness of a 20% price discount on fruit, vegetables, diet drinks and water, trialled in remote Australia to improve Indigenous health. PLoS ONE.

[28] Magnus A., Moodie M.L., Ferguson M., Cobiac L.J., Liberato S.C., Brimblecombe J. (2016). The economic feasibility of price discounts to improve diet in Australian Aboriginal remote communities. Aust. N. Z. J. Public Health.

[29] Ladapo J.A., Bogart L.M., Klein D.J., Cowgill B.O., Uyeda K., Binkle D.G., Stevens E.R., Schuster M.A. (2016). Cost and Cost-Effectiveness of Students for Nutrition and eXercise (SNaX). Acad. Pediatr..

[30] Allen K., Pearson-Stuttard J., Hooton W., Diggle P., Capewell S., O’Flaherty M. (2015). Potential of trans fats policies to reduce socioeconomic inequalities in mortality from coronary heart disease in England: Cost effectiveness modelling study. BMJ Br. Med. J..

[31] Gortmaker S.L., Wang Y.C., Long M.W., Giles C.M., Ward Z.J., Barrett J.L., Kenney E.L., Sonneville K.R., Sadaf Afzal A., Resch S.C. (2015). Three Interventions That Reduce Childhood Obesity Are Projected To Save More Than They Cost To Implement. Health Aff..

[32] Cobiac L.J., Vos T., Veerman J.L. (2010). Cost-effectiveness of interventions to promote fruit and vegetable consumption. PLoS ONE.

[33] Brimblecombe J., Ferguson M., Chatfield M.D., Liberato S.C., Gunther A., Ball K., Moodie M., Miles E., Magnus A., Mhurchu C.N. (2017). Effect of a price discount and consumer education strategy on food and beverage purchases in remote Indigenous Australia: A stepped-wedge randomised controlled trial. Lancet Public Health.

[34] Byker C.J., Farris A.R., Marcenelle M., Davis G.C., Serrano E.L. (2014). Food waste in a school nutrition program after implementation of new lunch program guidelines. J. Nutr. Educ. Behav..

[35] Gase L.N., McCarthy W.J., Robles B., Kuo T. (2014). Student receptivity to new school meal offerings: Assessing fruit and vegetable waste among middle school students in the Los Angeles Unified School District. Prev. Med..

[36] Blake M.R., Backholer K., Lancsar E., Boelsen-Robinson T., Mah C., Brimblecombe J., Zorbas C., Billich N., Peeters A. (2019). Investigating business outcomes of healthy food retail strategies: A systematic scoping review. Obes. Rev..

[37] McClung H.L., Ptomey L.T., Shook R.P., Aggarwal A., Gorczyca A.M., Sazonov E.S., Becofsky K., Weiss R., Das S.K. (2018). Dietary intake and physical activity assessment: Current tools, techniques, and technologies for use in adult populations. Am. J. Prev. Med..

[38] Taillie L.S., Grummon A.H., Fleischhacker S., Grigsby-Toussaint D.S., Leone L., Caspi C.E. (2017). Best practices for using natural experiments to evaluate retail food and beverage policies and interventions. Nutr. Rev..

[39] Odoms-Young A., Singleton C.R., Springfield S., McNabb L., Thompson T. (2016). Retail environments as a venue for obesity prevention. Curr. Obes. Rep..

[40] Rose D., Hutchinson P.L., Bodor J.N., Swalm C.M., Farley T.A., Cohen D.A., Rice J.C. (2009). Neighborhood food environments and body mass index: The importance of in-store contents. Am. J. Prev. Med..

[41] Ghosh-Dastidar B., Cohen D., Hunter G., Zenk S.N., Huang C., Beckman R., Dubowitz T. (2014). Distance to store, food prices, and obesity in urban food deserts. Am. J. Prev. Med..

[42] Tin S.T., Mhurchu C.N., Bullen C. (2007). Supermarket sales data: Feasibility and applicability in population food and nutrition monitoring. Nutr. Rev..

[43] Bandy L., Adhikari V., Jebb S., Rayner M. (2019). The use of commercial food purchase data for public health nutrition research: A systematic review. PLoS ONE.

[44] Timmins K.A., Green M.A., Radley D., Morris M.A., Pearce J. (2018). How has big data contributed to obesity research? A review of the literature. Int. J. Obes..

[45] Evans D. (2012). Beyond the throwaway society: Ordinary domestic practice and a sociological approach to household food waste. Sociology.

[46] Schanes K., Dobernig K., Gözet B. (2018). Food waste matters—A systematic review of household food waste practices and their policy implications. J. Clean. Prod..

[47] Graham-Rowe E., Jessop D.C., Sparks P. (2014). Identifying motivations and barriers to minimising household food waste. Resour. Conserv. Recycl..

[48] Garrone P., Melacini M., Perego A. (2014). Opening the black box of food waste reduction. Food Policy.

[49] Quested T.E., Parry A., Easteal S., Swannell R. (2011). Food and drink waste from households in the UK. Nutr. Bull..

[50] Williams M.B., Wang W., Taniguchi T., Salvatore A.L., Groover W.K., Wetherill M., Love C., Cannady T., Grammar M., Standridge J. (2020). Impact of a Healthy Retail Intervention on Fruits and Vegetables and Total Sales in Tribally Owned Convenience Stores: Findings From the THRIVE Study. Health Promot. Pract..

[51] Gemming L., Jiang Y., Swinburn B., Utter J., Mhurchu C.N. (2014). Under-reporting remains a key limitation of self-reported dietary intake: An analysis of the 2008/09 New Zealand Adult Nutrition Survey. Eur. J. Clin. Nutr..

[52] McMahon E., Wycherley T., O’Dea K., Brimblecombe J. (2017). A comparison of dietary estimates from the National Aboriginal and Torres Strait Islander Health Survey to food and beverage purchase data. Aust. N. Z. J. Public Health.

[53] Drummond M.F., Jefferson T. (1996). Guidelines for authors and peer reviewers of economic submissions to the BMJ. BMJ.

[54] Evers S., elle Goossens M., De Vet H., Van Tulder M., Banta D., Buxton M., Coyle D., Donaldson C., Drummond M., Elixhauser A. (2016). Criteria list for assessment of methodological quality of economic evaluations: Consensus on Health Economic Criteria The authors thank the following persons for their participation in the Delphi panel. Int. J. Technol. Assess. Health Care.

[55] Philips Z., Bojke L., Sculpher M., Claxton K., Golder S. (2006). Good practice guidelines for decision-analytic modelling in health technology assessment. Pharmacoeconomics.

[56] Watts R.D., Li I.W. (2019). Use of Checklists in Reviews of Health Economic Evaluations, 2010 to 2018. Value Health.

