# Supplementary material for: A Systematic Review of Economic Evaluations of Health-Promoting Food Retail-Based Interventions
Source: Int J Environ Res Public Health. 2021 Feb 2;18(3):1356. doi: 10.3390/ijerph18031356 (PMC7908088; doi:10.3390/ijerph18031356)
Supplement: Supplementary file 1 [file ijerph-18-01356-s001.pdf]

Table S1: PRISMA checklist

Section/topic	#	Checklist item	Reported on page #
**TITLE**			
Title	1	Identify the report as a systematic review, meta-analysis, or both.	1
**ABSTRACT**			
Structured summary	2	Provide a structured summary including, as applicable: background; objectives; data sources; study eligibility criteria, participants, and interventions; study appraisal and synthesis methods; results; limitations; conclusions and implications of key findings; systematic review registration number.	1
**INTRODUCTION**			
Rationale	3	Describe the rationale for the review in the context of what is already known.	1-2
Objectives	4	Provide an explicit statement of questions being addressed with reference to participants, interventions, comparisons, outcomes, and study design (PICOS).	1-2
**METHODS**			
Protocol and registration	5	Indicate if a review protocol exists, if and where it can be accessed (e.g., Web address), and, if available, provide registration information including registration number.	2
Eligibility criteria	6	Specify study characteristics (e.g., PICOS, length of follow-up) and report characteristics (e.g., years considered, language, publication status) used as criteria for eligibility, giving rationale.	2
Information sources	7	Describe all information sources (e.g., databases with dates of coverage, contact with study authors to identify additional studies) in the search and date last searched.	2
Search	8	Present full electronic search strategy for at least one database, including any limits used, such that it could be repeated.	2, Appendix B
Study selection	9	State the process for selecting studies (i.e., screening, eligibility, included in systematic review, and, if applicable, included in the meta-analysis).	2
Data collection process	10	Describe method of data extraction from reports (e.g., piloted forms, independently, in duplicate) and any processes	2-3

		for obtaining and confirming data from investigators.	
Data items	11	List and define all variables for which data were sought (e.g., PICOS, funding sources) and any assumptions and simplifications made.	2-3
Risk of bias in individual studies	12	Describe methods used for assessing risk of bias of individual studies (including specification of whether this was done at the study or outcome level), and how this information is to be used in any data synthesis.	Not applicable
Summary measures	13	State the principal summary measures (e.g., risk ratio, difference in means).	Not applicable
Synthesis of results	14	Describe the methods of handling data and combining results of studies, if done, including measures of consistency (e.g., I^2) for each meta-analysis.	2-3
Risk of bias across studies	15	Specify any assessment of risk of bias that may affect the cumulative evidence (e.g., publication bias, selective reporting within studies).	Not applicable
Additional analyses	16	Describe methods of additional analyses (e.g., sensitivity or subgroup analyses, meta-regression), if done, indicating which were pre-specified.	Not applicable
**RESULTS**			
Study selection	17	Give numbers of studies screened, assessed for eligibility, and included in the review, with reasons for exclusions at each stage, ideally with a flow diagram.	3-4
Study characteristics	18	For each study, present characteristics for which data were extracted (e.g., study size, PICOS, follow-up period) and provide the citations.	4-12
Risk of bias within studies	19	Present data on risk of bias of each study and, if available, any outcome level assessment (see item 12).	Not applicable
Results of individual studies	20	For all outcomes considered (benefits or harms), present, for each study: (a) simple summary data for each intervention group (b) effect estimates and confidence intervals, ideally with a forest plot.	12-18
Synthesis of results	21	Present results of each meta-analysis done, including confidence intervals and measures of consistency.	Not applicable
Risk of bias across studies	22	Present results of any assessment of risk of bias across studies (see Item 15).	Not applicable
Additional analysis	23	Give results of additional analyses, if done (e.g., sensitivity or subgroup analyses, meta-regression [see Item 16]).	Not

			applicable
**DISCUSSION**			
Summary of evidence	24	Summarize the main findings including the strength of evidence for each main outcome; consider their relevance to key groups (e.g., healthcare providers, users, and policy makers).	19-21
Limitations	25	Discuss limitations at study and outcome level (e.g., risk of bias), and at review-level (e.g., incomplete retrieval of identified research, reporting bias).	21
Conclusions	26	Provide a general interpretation of the results in the context of other evidence, and implications for future research.	21
**FUNDING**			
Funding	27	Describe sources of funding for the systematic review and other support (e.g., supply of data); role of funders for the systematic review.	22

Table S2: List of search terms used for the database searches

Search concept	Search terms
Concept 1	1. value N2 money<br>2. "health economics" or "cost containment" or "health resource* allocat*" or "health resource* utili?at*" or "evaluation stud*"<br>3. economic* N2 (study or studies or costing or model* or evaluat* or analy* or pricing)<br>4. S1 OR S2 OR S3 OR S4<br>supermarket* or hypermarket* or restaurant* or café or cafeteria* or "milk bar*" or "tuck shop*" or delicatessen* or deli or delis or "food court*"
Concept 2	6. supermarket* or hypermarket* or restaurant* or café or cafeteria* or "milk bar*" or "tuck shop*" or delicatessen* or deli or delis or "food court*"<br>7. (food* or grocer* or corner or convenience) N3 (store* or retail* or market or markets)<br>8. "in store" or "in-store"<br>9. "store based" or "store-based"<br>10. "point-of-purchase" or "point of purchase"<br>11. S6 OR S7 OR S8 OR S9 OR S10
Concept 3	12. intervention* or promot* or program* or marketing<br>13. food* N3 (supply* or supplie* or industr* or accessibilit* or environment* or price* or pricing or cost* or adverti* or promot*)<br>14. S12 OR S13
Concept 4	15. diet* N3 (pattern* or chang* or intake*)<br>16. (health* or nutrition*) N2 status<br>17. BMI or "body mass" or overweight or "over-weight" or "body fat" or "body composition" or obes* or adipos* or weight<br>18. food* N3 (purchas* or consume or consumed or consumption or consumes or sale* or choice* or intake*)<br>19. (behavio* or habit*) N3 (chang* or modif* or food* or feeding or eating)<br>20. (energy or calorie*) N3 (intake* or chang* or pattern*)<br>21. S15 OR S16 OR S17 OR S18 OR S19 OR S20
Combined searches	22. S5 AND S11 AND S14<br>23. S5 AND S11 AND S21<br>24. S22 OR S23

Table S3: List of Medical Subject Headings terms used for the database searches

Database	Concept	MeSH terms
Academic Search Complete	1	(((DE "MEDICAL economics")) OR (DE "COST effectiveness")) OR (DE "COST effectiveness of health promotion")
	2	((DE "SUPERMARKETS") OR (DE "HYPERMARKETS")) OR (DE "COFFEEHOUSES")) OR (DE "CAFETERIAS")) OR (DE "DELICATESSENS")) OR (DE "FOOD courts")) OR (DE "RETAIL stores")) OR (DE "BRANCH stores")) OR (DE "CHAIN stores")) OR (DE "CONVENIENCE stores")) OR (DE "SELF-service stores")) OR (DE "COMBINATION stores")) OR (DE "NATURAL food stores")) OR (DE "COLLEGE stores")) OR (DE "GENERAL stores")) OR (DE "SPECIALTY food stores")) OR (DE "ONE-stop shopping")) OR (DE "SERVICE stations")) OR (DE "HOTEL stores")) OR (DE "STORE-within-a-store")) OR (DE "STOREFRONTS")) OR (DE "TEMPORARY stores")) OR (DE "RETAIL franchises")) OR (DE "GROCERY shopping")) OR (DE "GROCERIES"))) OR (DE "SCHOOL lunchrooms, cafeterias, etc.")) OR (DE "FOOD service in universities & colleges"))) OR (DE "RETAIL store health clinics"))) OR (DE "RESTAURANTS" OR DE "AIRPORT restaurants" OR DE "BAGEL & doughnut shops" OR DE "BARBECUE restaurants" OR DE "BREWPUBS" OR DE "BUFFET restaurants" OR DE "CHAIN restaurants" OR DE "COFFEE shops" OR DE "COFFEEHOUSES" OR DE "CYBERCAFES" OR DE "DELICATESSENS" OR DE "DINERS (Restaurants)" OR DE "DISCRIMINATION in restaurants" OR DE "DRIVE-through restaurants" OR DE "ENTERTAINMENT in restaurants" OR DE "ETHNIC restaurants" OR DE "FAST food restaurants" OR DE "FLOATING restaurants" OR DE "FULL service restaurants" OR DE "HOTEL restaurants" OR DE "ICE cream parlors" OR DE "INDEPENDENT restaurants" OR DE "KOSHER restaurants" OR DE "LGBT restaurants" OR DE "LIMITED-service restaurants" OR DE "MACROBIOTIC restaurants" OR DE "NATURAL food restaurants" OR DE "PIZZERIAS" OR DE "RESTAURANTS as an investment" OR DE "REVOLVING restaurants" OR DE "ROADSIDE restaurants" OR DE "SANDWICH shops" OR DE "SCHOOL lunchrooms, cafeterias, etc." OR DE "SEAFOOD restaurants" OR DE "SMOKING in restaurants" OR DE "STEAK houses" OR DE "TAKE-out restaurants" OR DE "TEAROOMS" OR DE "THEME restaurants" OR DE "UNDERGROUND restaurants" OR DE "VEGETARIAN restaurants" OR DE "WOMEN'S restaurants")
	3	(((((((((((((((DE "PROMOTIONS") OR (DE "MARKETING")) OR (DE "SALES promotion")) OR (DE "PROMOTIONAL products")) OR (DE "POINT-of-sale advertising")) OR (DE "ADVERTISING")) OR (DE "SPECIAL sales")) OR (DE "MARKDOWNS (Retail industry)")) OR (DE "ADVERTISING")) OR (DE "POINT-of-sale advertising")) OR (DE "BAKED product advertising")) OR (DE "GENERIC product advertising")) OR (DE "PASTA product advertising")) OR (DE "PRODUCT demonstrations")) OR (DE "SALES presentations")) OR (DE "ADVERTISING campaigns") OR (((DE "FOOD supply") OR (DE "FOOD industry")) AND (DE "AQUACULTURE industry" OR DE "FOOD advertising")) OR (DE "FOOD prices")

	4	(((((((((((((((((((DE "DIET") OR (DE "FOOD portions")) OR (DE "FOOD habits")) OR (DE "FOOD preferences")) OR (DE "MEAL frequency")) OR (DE "DRINKING behavior")) OR (DE "HEALTH")) OR (DE "HEALTH behavior")) OR (DE "QUALITY of life")) OR (DE "NUTRITIONAL assessment")) OR (DE "BODY mass index" OR DE "BODY weight")) OR (DE "WEIGHTS & measures")) OR (DE "OBESITY")) OR (DE "OVERWEIGHT persons")) OR (DE "OVERWEIGHT teenagers")) OR (DE "WEIGHT loss")) OR (DE "WEIGHT gain")) OR (DE "BODY composition")) OR (DE "HUMAN body composition")) OR (DE "WAIST circumference") OR ((DE "FOOD consumption") OR (DE "BEHAVIOR modification")) OR (DE "REDUCING diets")
Business Source Complete	1	((DE "MEDICAL economics") OR (DE "COST effectiveness")) OR (DE "COST effectiveness of health promotion")
	2	((((((((((((((((((((((((((((((((DE "SUPERMARKETS") OR (DE "COMBINATION stores")) OR (DE "NATURAL food stores")) OR (DE "SELF-service stores")) OR (DE "GENERAL stores")) OR (DE "HYPERMARKETS")) OR (DE "CONVENIENCE stores")) OR (DE "SERVICE station convenience stores")) OR (DE "CHAIN stores")) OR (DE "ONE-stop shopping")) OR (DE "RETAIL stores")) OR (DE "SPECIALTY stores")) OR (DE "RESTAURANTS")) OR (DE "AIRPORT restaurants")) OR (DE "BAGEL & doughnut shops")) OR (DE "BUFFET restaurants")) OR (DE "CHAIN restaurants")) OR (DE "COFFEE shops")) OR (DE "DELICATESSENS")) OR (DE "LIMITED-service restaurants")) OR (DE "PIZZERIAS")) OR (DE "SANDWICH shops")) OR (DE "TAKE-out restaurants")) OR (DE "NON-alcoholic beverage bars")) OR (DE "INDEPENDENT restaurants")) OR (DE "EMPLOYEE food service")) OR (DE "FOOD courts")) OR (DE "MEAL assembly stores")) OR (DE "STADIUMS -- Food service")) OR (DE "GROCERY industry")) OR (DE "INDEPENDENT grocery stores")) OR (DE "ETHNIC markets")) OR (DE "BUSINESS food service")
	3	((((((((DE "MARKETING") OR (DE "ADVERTISING")) AND (DE "SALES promotion" OR DE "ADVERTISING campaigns")) OR (DE "MARKDOWNS (Retail industry)")) OR (DE "SPECIAL sales")) OR (DE "PRODUCT demonstrations")) OR (DE "SALES presentations")) OR (DE "ADVERTISING campaigns") OR (DE "COUPONS (Retail trade)")) OR (((((DE "FOOD supply") OR (DE "FOOD industry")) OR (DE "FOOD labeling")) OR (DE "FOOD packaging")) OR (DE "FOOD prices"))
	4	(((DE "GROCERY shopping") OR (DE "FOOD consumption statistics")) OR (DE "CONSUMER behavior")) OR (DE "CONSUMER preferences")
CINAHL Complete	1	(MH "Costs and Cost Analysis") OR (MH "Cost Benefit Analysis") OR (MH "Health Resource Allocation")
	2	(MH "Restaurants")
	3	(MH "Marketing+") OR (MH "Advertising") OR (MH "Food Supply") OR (MH "Food Industry")
	4	(MH "Eating Behavior+") OR (MH "Habits+") OR (MH "Health Status") OR (MH "Nutritional Status") OR (MH "Nutritional Status: Body Mass (Iowa NOC)") OR (MH "Nutritional Status: Energy (Iowa NOC)") OR (MH "Nutritional Status: Nutrient Intake (Iowa NOC)") OR (MH "Nutritional Status: Food & Fluid Intake (Iowa NOC)") OR (MH "Nutritional Status (Iowa NOC)") OR (MH "Body Mass Index") OR (MH "Body Size") OR (MH "Body

		Weight+") OR (MH "Waist Circumference") OR (MH "Fat Free Mass") OR (MH "Obesity") OR (MH "Weight Gain+") OR (MH "Weight Loss") OR (MH "Body Weight Changes+") OR (MH "Energy Intake") OR (MH "Food Intake+") OR (MH "Diet, Reducing")
EconLit	1	((ZE "economics of health (including medical subsidy programs)")) or ((ZE "economics of health including medical subsidy programs"))
	2	((ZE "retail food")) or ((ZE "retailing"))
	3	(((((((((ZE "marketing")) or ((ZE "marketing and advertising"))) or ((ZE "marketing and advertising: general"))) or ((ZE "marketing and advertising: government policy and regulation"))) or ((ZE "marketing and advertising: other"))) or ((ZE "marketing: general"))) or ((ZE "marketing: other distribution policies"))) or ((ZE "marketing: price policies"))) or ((ZE "marketing: product policies")) OR ((ZE "food; beverages; cosmetics; tobacco; wine and spirits")) or ((ZE "food subsidies"))
	4	(ZE "food consumption")
Global Health	1	((((DE "economic analysis") OR (DE "cost analysis")) OR (DE "cost benefit analysis")) OR (DE "cost effectiveness analysis")) OR (DE "economic evaluation")
	2	(((((((((((DE "supermarkets") OR (DE "hypermarkets")) OR (DE "small shops")) OR (DE "restaurants" OR DE "fast food restaurants" OR DE "speciality restaurants" OR DE "takeout restaurants" OR DE "theme restaurants")) OR (DE "theme restaurants")) OR (DE "takeout restaurants")) OR (DE "speciality restaurants")) OR (DE "fast food restaurants")) OR (DE "cafes")) OR (DE "cafeterias")) OR (DE "stores")) OR (DE "shops")) OR (DE "street markets")
	3	(((((((DE "sales promotion") OR (DE "advertising")) OR (DE "food advertising")) OR (DE "discounts")) OR (DE "food supply")) OR (DE "food industry")) OR (DE "food prices")) OR (DE "food costs")
	4	(((((((((DE "weight control") OR (DE "weight reduction")) OR (DE "weight losses")) OR (DE "weight gain")) OR (DE "overweight")) OR (DE "obesity")) OR (DE "body fat")) OR (DE "body mass index")) OR (DE "body composition")) OR (DE "body lean mass") OR (((((((((DE "food purchasing") OR (DE "food consumption")) OR (DE "consumption patterns")) OR (DE "eating patterns")) OR (DE "meal patterns")) OR (DE "feeding behaviour")) OR (DE "food intake")) OR (DE "caloric intake")) OR (DE "energy intake")) OR (DE "food preferences" OR (DE "feeding preferences")) OR (DE "feeding habits")
Health Business Elite	1	(((DE "RESOURCE allocation") OR (DE "MEDICAL economics")) OR (DE "COST effectiveness of health promotion")) OR (DE "COST effectiveness")
	2	((DE "SUPERMARKETS") OR (DE "COMBINATION stores")) OR (DE "NATURAL food stores")) OR (DE "SELF-service stores")) OR (DE "HYPERMARKETS")) OR (DE "RETAIL stores")) OR (DE "CONVENIENCE stores")) OR (DE "BRANCH stores")) OR (DE "CHAIN stores")) OR (DE "SPECIALTY stores")) OR (DE "RETAIL franchises")) OR (DE "RESTAURANTS")) OR (DE "AIRPORT

		restaurants")) OR (DE "BAGEL & doughnut shops")) OR (DE "BARBECUE restaurants")) OR (DE "BUFFET restaurants")) OR (DE "CHAIN restaurants")) OR (DE "COFFEE shops")) OR (DE "COFFEEHOUSES")) OR (DE "DELICATESSENS")) OR (DE "DINERS (Restaurants)")) OR (DE "DRIVE-through restaurants")) OR (DE "ETHNIC restaurants")) OR (DE "FAST food restaurants")) OR (DE "FULL service restaurants")) OR (DE "HOTEL restaurants")) OR (DE "INDEPENDENT restaurants")) OR (DE "KOSHER restaurants")) OR (DE "LGBT restaurants")) OR (DE "LIMITED-service restaurants")) OR (DE "MACROBIOTIC restaurants")) OR (DE "NATURAL food restaurants")) OR (DE "PIZZERIAS")) OR (DE "REVOLVING restaurants")) OR (DE "ROADSIDE restaurants")) OR (DE "SANDWICH shops")) OR (DE "SCHOOL lunchrooms, cafeterias, etc.")) OR (DE "SEAFOOD restaurants")) OR (DE "STEAK houses")) OR (DE "TAKE-out restaurants")) OR (DE "THEME restaurants")) OR (DE "UNDERGROUND restaurants")) OR (DE "VEGETARIAN restaurants")) OR (DE "CAFETERIAS")) OR (DE "ONE-stop shopping")) OR (DE "SERVICE station convenience stores")
	3	((DE "MARKETING") OR (DE "PRODUCT demonstrations")) OR (((DE "SALES promotion") OR (DE "POINT-of-sale advertising")) OR (DE "PROMOTIONAL products")) OR (DE "ADVERTISING campaigns") OR ((((DE "FOOD supply") OR (DE "FOOD industry")) OR (DE "GROCERY industry")) OR (DE "FOOD prices")) OR (DE "FOOD advertising")
	4	(((((((((((((((((((((((DE "HEALTH status indicators") OR (DE "NUTRITIONAL status"))) OR (DE "OBESITY")) OR (DE "BODY weight")) OR (DE "BODY size")) OR (DE "BODY mass index")) OR (DE "WEIGHT gain")) OR (DE "WEIGHT loss")) OR (DE "BODY composition")) OR (DE "OBESITY in adolescence")) OR (DE "OBESITY in children")) OR (DE "OBESITY in men"))) OR (DE "WEIGHT loss")) OR (DE "OBESITY in women") OR ((DE "FOOD consumption") OR (DE "FOOD preferences")) OR (DE "FOOD habits")
Health Policy Reference Center	1	(((DE "MEDICAL economics") OR (DE "RESOURCE allocation")) OR (DE "COST effectiveness")) OR (DE "COST effectiveness of health promotion")
	2	(((DE "SUPERMARKETS") OR (DE "COMBINATION stores")) OR (DE "NATURAL food stores")) OR (DE "SELF-service stores")) OR (DE "RETAIL stores")) OR (DE "BRANCH stores")) OR (DE "CHAIN stores")) OR (DE "COLLEGE stores")) OR (DE "COMPANY stores")) OR (DE "CONVENIENCE stores")) OR (DE "GENERAL stores")) OR (DE "SPECIALTY stores")) OR (DE "STORE-within-a-store")) OR (DE "ONE-stop shopping")) OR (DE "ETHNIC markets")) OR (DE "INDEPENDENT grocery stores")) OR (DE "CONVENIENCE stores")) OR (DE "SPECIALTY food stores")) OR (DE "HYPERMARKETS")) OR (DE "RESTAURANTS")) OR (DE "AIRPORT restaurants")) OR (DE "BAGEL & doughnut shops")) OR (DE "BARBECUE restaurants")) OR (DE "BUFFET restaurants")) OR (DE "CHAIN restaurants")) OR (DE "COFFEE shops")) OR (DE "COFFEEHOUSES")) OR (DE "DELICATESSENS")) OR (DE "DINERS (Restaurants)")) OR (DE "DRIVE-through restaurants")) OR (DE "ETHNIC restaurants")) OR (DE "FAST food restaurants")) OR (DE "FLOATING restaurants")) OR (DE "FULL service restaurants")) OR (DE "HOTEL restaurants")) OR (DE

		"INDEPENDENT restaurants")) OR (DE "KOSHER restaurants")) OR (DE "LGBT restaurants")) OR (DE "LIMITED-service restaurants")) OR (DE "MACROBIOTIC restaurants")) OR (DE "NATURAL food restaurants")) OR (DE "PIZZERIAS")) OR (DE "REVOLVING restaurants")) OR (DE "ROADSIDE restaurants")) OR (DE "SANDWICH shops")) OR (DE "SCHOOL lunchrooms, cafeterias, etc.")) OR (DE "SEAFOOD restaurants")) OR (DE "STEAK houses")) OR (DE "TAKE-out restaurants")) OR (DE "THEME restaurants")) OR (DE "THEME restaurants")) OR (DE "UNDERGROUND restaurants")) OR (DE "VEGETARIAN restaurants")) OR (DE "CAFETERIAS")) OR (DE "FOOD courts")
	3	((((((DE "MARKETING") OR (DE "ADVERTISING")) OR (DE "PRODUCT demonstrations")) OR (DE "SALES presentations")) OR (DE "SALES promotion")) OR (DE "ADVERTISING campaigns")) OR (DE "POINT-of-sale advertising") OR (((DE "FOOD supply") OR (DE "FOOD industry")) OR (DE "FOOD prices") OR (DE "FOOD advertising")
	4	((((((((((((DE "HEALTH status indicators") OR (DE "NUTRITIONAL status")) OR (DE "WEIGHT loss")) OR (DE "BODY weight")) OR (DE "OBESITY")) OR (DE "WEIGHT gain")) OR (DE "BODY mass index")) OR (DE "BODY composition")) OR (DE "HUMAN body composition")) OR (DE "WAIST circumference")) OR (DE "OBESITY in adolescence")) OR (DE "OBESITY in children")) OR (DE "OBESITY in women") OR ((DE "FOOD consumption") OR (DE "FOOD preferences")) OR (DE "FOOD habits")
Health Source: Nursing/Academic Edition	1	(((DE "MEDICAL economics") OR (DE "RESOURCE allocation")) OR (DE "COST effectiveness")) OR (DE "COST effectiveness of health promotion")
	2	(((DE "SUPERMARKETS") OR (DE "HYPERMARKETS")) OR (DE "RESTAURANTS")) OR (DE "AIRPORT restaurants")) OR (DE "BAGEL & doughnut shops")) OR (DE "BARBECUE restaurants")) OR (DE "BUFFET restaurants")) OR (DE "CHAIN restaurants")) OR (DE "COFFEE shops")) OR (DE "COFFEEHOUSES")) OR (DE "DELICATESSENS")) OR (DE "DINERS (Restaurants)")) OR (DE "DRIVE-through restaurants")) OR (DE "ETHNIC restaurants")) OR (DE "FAST food restaurants")) OR (DE "FLOATING restaurants")) OR (DE "FULL service restaurants")) OR (DE "HOTEL restaurants")) OR (DE "INDEPENDENT restaurants")) OR (DE "KOSHER restaurants")) OR (DE "LGBT restaurants")) OR (DE "LIMITED-service restaurants")) OR (DE "MACROBIOTIC restaurants")) OR (DE "NATURAL food restaurants")) OR (DE "PIZZERIAS")) OR (DE "REVOLVING restaurants")) OR (DE "ROADSIDE restaurants")) OR (DE "SANDWICH shops")) OR (DE "SCHOOL lunchrooms, cafeterias, etc.")) OR (DE "SEAFOOD restaurants")) OR (DE "STEAK houses")) OR (DE "TAKE-out restaurants")) OR (DE "THEME restaurants")) OR (DE "UNDERGROUND restaurants")) OR (DE "VEGETARIAN restaurants")) OR (DE "CAFETERIAS")) OR (DE "FOOD courts")) OR (DE "RETAIL stores")) OR (DE "BRANCH stores")) OR (DE "CHAIN stores")) OR (DE "COLLEGE stores")) OR (DE "COMBINATION stores")) OR (DE "COMPANY stores")) OR (DE "CONVENIENCE stores")) OR (DE "GENERAL stores")) OR (DE "SELF-service stores")) OR (DE "SPECIALTY

		stores")) OR (DE "STORE-within-a-store")) OR (DE "ONE-stop shopping")) OR (DE "GROCERY industry")) OR (DE "SPECIALTY food stores")) OR (DE "SERVICE station convenience stores")
	3	(((((DE "MARKETING") OR (DE "PRODUCT demonstrations")) OR (DE "SALES promotion")) OR (DE "ADVERTISING")) OR (DE "ADVERTISING campaigns")) OR (DE "POINT-of-sale advertising") OR (((DE "FOOD supply") OR (DE "FOOD industry")) OR (DE "FOOD prices")) OR (DE "FOOD advertising")
	4	((((((((((((((DE "HEALTH status indicators") OR (DE "NUTRITIONAL status")) OR (DE "WEIGHT loss")) OR (DE "OBESITY")) OR (DE "WEIGHT gain")) OR (DE "BODY weight")) OR (DE "BODY mass index")) OR (DE "BODY size")) OR (DE "WEIGHTS & measures")) OR (DE "OBESITY in adolescence")) OR (DE "OBESITY in children")) OR (DE "OBESITY in women")) OR (DE "BODY composition")) OR (DE "HUMAN body composition")) OR (DE "LEAN body mass")) OR (DE "WAIST circumference") OR ((DE "FOOD consumption") OR (DE "FOOD preferences")) OR (DE "FOOD habits")
MEDLINE Complete	1	(MH "Health Care Sector") OR (MH "Cost-Benefit Analysis") OR (MH "Resource Allocation") OR (MH "Health Care Rationing")
	2	(MH "Restaurants")
	3	(MH "Marketing") OR (MH "Food Supply") OR (MH "Food Industry")
	4	(MH "Feeding Behavior") OR (MH "Health Status") OR (MH "Nutritional Status") OR (MH "Overweight") OR (MH "Body Mass Index") OR (MH "Obesity") OR (MH "Pediatric Obesity") OR (MH "Body Fat Distribution") OR (MH "Adiposity") OR (MH "Food Preferences") OR (MH "Feeding Behavior") OR (MH "Eating") OR (MH "Diet+") OR (MH "Energy Intake+")
PsycINFO	1	((DE "Health Care Economics") OR (DE "Costs and Cost Analysis")) OR (DE "Resource Allocation")
	2	(DE "Fast Food") OR (DE "Retailing")
	3	DE "Marketing"
	4	((((((((((DE "Health Status") OR (DE "Body Mass Index")) OR (DE "Body Weight")) OR (DE "Obesity")) OR (DE "Overweight")) OR (DE "Body Fat")) OR (DE "Food Intake")) OR (DE "Eating Behavior")) OR (DE "Weight Control")) OR (DE "Weight Gain")) OR (DE "Weight Loss")
SocINDEX with Full Text	1	((DE "MEDICAL economics") OR (DE "RESOURCE allocation")) OR (DE "COST effectiveness")
	2	((((DE "RETAIL industry") OR (DE "MARKETS")) OR (DE "ETHNIC markets")) OR (DE "FARMERS' markets")) OR (DE "COOPERATIVE societies")
	3	((((DE "MARKETING") OR (DE "ADVERTISING")) OR (DE "FOOD supply")) OR (DE "FOOD industry")) OR (DE "COST & standard of living")

	4	(((((((DE "HEALTH status indicators") OR (DE "NUTRITIONAL status")) OR (DE "BODY weight")) OR (DE "OBESITY")) OR (DE "OBESITY in adolescence")) OR (DE "OBESITY in children")) OR (DE "FOOD consumption")) OR (DE "FOOD habits")
Embase	1	'health economics'/de OR 'economic evaluation'/exp OR 'resource allocation'/exp
	2	'restaurant'/exp
	3	'intervention study'/exp OR 'direct-to-consumer advertizing'/exp OR 'takeaway (food)'/exp OR 'food environment'/exp OR 'food industry'/de
	4	'dietary pattern'/exp OR 'dietary intake'/de OR 'health status'/de OR 'nutritional status'/de OR 'body mass'/de OR 'weight, mass and size'/de OR 'body size'/exp OR 'body weight'/de OR 'waist circumference'/exp OR 'body composition'/exp OR 'obesity'/de OR 'adolescent obesity'/exp OR 'childhood obesity'/exp OR 'maternal obesity'/exp OR 'food intake'/de OR 'energy consumption'/exp OR 'portion size'/exp OR 'food choice'/de OR 'feeding behavior'/de OR 'eating habit'/exp OR 'food preference'/exp OR 'caloric intake'/exp

Table S4: CHEERS Checklist of included studies

Section/item	Item No	Recommendation	Trial-based evaluations			Model-based evaluations				
			CEA			CEA & CUA		CUA		
			Ball et al. 2016 [25]	Ladapo et al. 2016 [29]	Le et al. 2016 [26]	Gortmaker et al. 2015 [31]	Allen et al. 2015 [30]	Cobiac et al. [32]	Magnus et al. 2016 [28]	Magnus et al. 2018 [27]
**Title and abstract**										
Title	1	Identify the study as an economic evaluation or use more specific terms such as "cost-effectiveness analysis", and describe the interventions compared.	×	✓	✓	×	×	✓	×	×
Abstract	2	Provide a structured summary of objectives, perspective, setting, methods (including study design and inputs), results (including base case and uncertainty analyses), and conclusions.	×	×	✓	×	×	✓	×	×
**Introduction**										
Background and objectives	3	Provide an explicit statement of the broader context for the study. Present the study question and its relevance for health policy or practice decisions.	✓	✓	✓	✓	✓	✓	✓	✓
**Methods**										
Target population and subgroups	4	Describe characteristics of the base case population and subgroups analysed, including why they were chosen.	✓	✓	✓	✓	✓	✓	✓	✓
Setting and location	5	State relevant aspects of the system(s) in which the decision(s) need(s) to be made.	×	✓	✓	✓	✓	✓	×	×
Study perspective	6	Describe the perspective of the study and relate this to the costs being	✓	✓	✓	✓	✓	✓	✓	✓

		evaluated.								
Comparators	7	Describe the interventions or strategies being compared and state why they were chosen.	✓	✓	✓	✗	✓	✓	✗	✓
Time horizon	8	State the time horizon(s) over which costs and consequences are being evaluated and say why appropriate.	✓	✓	✓	✓	✗	✓	✓	✗
Discount rate	9	Report the choice of discount rate(s) used for costs and outcomes and say why appropriate.	NA	NA	NA	✓	✓	✓	✓	✓
Choice of health outcomes	10	Describe what outcomes were used as the measure(s) of benefit in the evaluation and their relevance for the type of analysis performed.	✓	✓	✓	✓	✗	✓	✓	✓
Measurement of effectiveness	11a	*Single study-based estimates:* Describe fully the design features of the single effectiveness study and why the single study was a sufficient source of clinical effectiveness data.	✓	✓	✓	NA	NA	✓	NA	✓
	11b	*Synthesis-based estimates:* Describe fully the methods used for identification of included studies and synthesis of clinical effectiveness data.	NA	NA	NA	✓	✗	NA	✓	NA
Measurement and valuation of preference based outcomes	12	If applicable, describe the population and methods used to elicit preferences for outcomes.	NA	NA	NA	NA	✗	✓	NA	✗
Estimating resources and costs	13a	*Single study-based economic evaluation:* Describe approaches used to estimate resource use associated with the alternative interventions. Describe primary or secondary research methods for valuing each resource item in terms of its unit cost. Describe any adjustments made to approximate to opportunity costs.	✗	✓	✓	NA	NA	NA	NA	✓
	13b	*Model-based economic evaluation:* Describe approaches and data sources used to estimate resource use associated with model health states. Describe primary or secondary research methods for valuing each resource item in terms of its unit cost. Describe any adjustments made to approximate to opportunity costs.	NA	NA	NA	✗	✓	✓	✓	NA
Currency, price date, and conversion	14	Report the dates of the estimated resource quantities and unit costs. Describe methods for adjusting estimated unit costs to the year of reported costs if necessary. Describe methods for converting costs into a common currency base and the exchange rate.	✓	✓	✓	✓	✓	✓	✓	✓

Choice of model	15	Describe and give reasons for the specific type of decision-analytical model used. Providing a figure to show model structure is strongly recommended.	NA	NA	NA	✓	✓	✓	×	×
Assumptions	16	Describe all structural or other assumptions underpinning the decision-analytical model.	NA	NA	NA	✓	✓	✓	✓	✓
Analytical methods	17	Describe all analytical methods supporting the evaluation. This could include methods for dealing with skewed, missing, or censored data; extrapolation methods; methods for pooling data; approaches to validate or make adjustments (such as half cycle corrections) to a model; and methods for handling population heterogeneity and uncertainty.	✓	×	✓	✓	✓	✓	✓	×
**Results**										
Study parameters	18	Report the values, ranges, references, and, if used, probability distributions for all parameters. Report reasons or sources for distributions used to represent uncertainty where appropriate. Providing a table to show the input values is strongly recommended.	×	×	✓	✓	✓	✓	×	✓
Incremental costs and outcomes	19	For each intervention, report mean values for the main categories of estimated costs and outcomes of interest, as well as mean differences between the comparator groups. If applicable, report incremental cost-effectiveness ratios.	✓	✓	✓	×	×	✓	✓	✓
Characterising uncertainty	20a	*Single study-based economic evaluation:* Describe the effects of sampling uncertainty for the estimated incremental cost and incremental effectiveness parameters, together with the impact of methodological assumptions (such as discount rate, study perspective).	×	✓	✓	NA	NA	NA	NA	✓
	20b	*Model-based economic evaluation:* Describe the effects on the results of uncertainty for all input parameters, and uncertainty related to the structure of the model and assumptions.	NA	NA	NA	✓	✓	✓	✓	NA
Characterising heterogeneity	21	If applicable, report differences in costs, outcomes, or cost- effectiveness that can be explained by variations between subgroups of patients with different baseline characteristics or other observed variability in effects that are not reducible by more information.	✓	×	×	×	✓	×	×	×
**Discussion**										
Study findings, limitations,	22	Summarise key study findings and describe how they support the conclusions reached. Discuss limitations and the generalisability of the	✓	✓	✓	✓	×	✓	✓	×

generalisability, and current knowledge		findings and how the findings fit with current knowledge.								
**Other**										
Source of funding	23	Describe how the study was funded and the role of the funder in the identification, design, conduct, and reporting of the analysis. Describe other non-monetary sources of support.	✓	×	✓	✓	✓	✓	×	✓
Conflicts of interest	24	Describe any potential for conflict of interest of study contributors in accordance with journal policy. In the absence of a journal policy, we recommend authors comply with International Committee of Medical Journal Editors recommendations.	✓	✓	✓	×	✓	✓	✓	✓
**Percentage of compliance**			**70%**	**75%**	**95%**	**70%**	**67%**	**96%**	**65%**	**65%**

Notes: × not reported; ✓ reported; NA: not applicable
